# How do environmental governance processes shape evaluation of outcomes by stakeholders? A causal pathways approach

**DOI:** 10.1371/journal.pone.0185375

**Published:** 2017-09-25

**Authors:** Ryan Plummer, Angela Dzyundzyak, Julia Baird, Örjan Bodin, Derek Armitage, Lisen Schultz

**Affiliations:** 1 Environmental Sustainability Research Centre, Brock University, St. Catharines, Ontario, Canada; 2 Stockholm Resilience Centre, Stockholm University, Stockholm, Sweden; 3 School of Environment, Resources and Sustainability, University of Waterloo, Waterloo, Ontario, Canada; Sveriges lantbruksuniversitet, SWEDEN

## Abstract

Multi-stakeholder environmental management and governance processes are essential to realize social and ecological outcomes. Participation, collaboration, and learning are emphasized in these processes; to gain insights into how they influence stakeholders’ evaluations of outcomes in relation to management and governance interventions we use a path analysis approach to examine their relationships in individuals in four UNESCO Biosphere Reserves. We confirm a model showing that participation in more activities leads to greater ratings of process, and in turn, better evaluations of outcomes. We show the effects of participation in activities on evaluation of outcomes appear to be driven by learning more than collaboration. Original insights are offered as to how the evaluations of outcomes by stakeholders are shaped by their participation in activities and their experiences in management and governance processes. Understanding stakeholder perceptions about the processes in which they are involved and their evaluation of outcomes is imperative, and influences current and future levels of engagement. As such, the evaluation of outcomes themselves are an important tangible product from initiatives. Our research contributes to a future research agenda aimed at better understanding these pathways and their implications for engagement in stewardship and ultimately social and ecological outcomes, and to developing recommendations for practitioners engaged in environmental management and governance.

## Introduction

We are confronted with the challenge of stewardship in the Anthropocene where humans are a primary driver of environmental change [1,2]. In this era, stakeholder participation, collaboration and learning are important features of management and governance approaches needed to enhance social and ecological outcomes [3,4,5]. Yet, to the best of our knowledge it has not been empirically proven that these features lead to enhanced evaluation of outcomes nor are their interactions well understood. Empirically linking management and governance interventions to outcomes is a pressing need in natural resource settings [6], evidence-based conservation [7], and environmental decision-making [8,9].

The importance of forging connections among participation, learning and collaboration is reflected in diverse resource management and governance approaches [2, 10–13]. For example, adaptive co-management or adaptive governance approaches emphasize the importance of stakeholder participation in decision making activities, as well as a need to harness the power of collaboration, draw upon diverse knowledge types, and facilitate the institutional flexibility to respond to ecosystem change [12–14]. However, empirically validating the relationships among these features and in combination with evaluating outcomes is complicated and contested. Gaining insights on these variables, their connections and contributions to evaluating outcomes in adaptive and collaborative management processes in a systematic way is an identified knowledge gap and an ongoing need [15–18] to advance scholarly research and to support decision making in environmental stewardship practice. Thus, our research focuses on establishing and understanding the causal relationships among participation, social processes that occur in governance, and evaluations of outcomes, using the individual stakeholder perspective.

### Stakeholder participation

While greater stakeholder participation is not appropriate under all circumstances [19,20], arguments persist that participation is linked to advancing deliberative decision making, increasing efficiency, and enhancing effectiveness [20–23]. Improving stakeholder involvement in making decisions about the environment is now embedded in policy discourses across scales to enhance governance [23, 19]. However, claims about stakeholder participation and social and ecological outcomes are often presumed, but rarely proven ([23, 24], see [21] for an exception). A synthesis of studies that do evaluate participation [23] (e.g., [25–27]) suggests improvements in quality of decisions are strongly contingent upon the qualities of the associated process.

### Collaboration

Multi-party (i.e., collaboration-based) solutions have provided an avenue to overcome impasses and tackle complex problems. Engaging diverse individuals in an interactive and iterative problem-solving process may lead to better decisions, increase implementation and build capacity for the future [3,4]. Extensive research over the past decades has documented a wide range of cases of multi-party collaboration relating to the environment. This research has offered valuable insights into qualities of collaboration which influence success, such as transparency [28], social capital [29], willingness to compromise [6] and leadership [6, 30]. However, meta-analyses and reviews of collaborative environmental governance have revealed a mix of positive and negative outcomes [31, 32].

### Learning

The shift towards ecosystem-based and adaptive management has stressed the need to learn from large-scale experiments and ‘learn by doing’ [33, 34]. Learning has become a normative goal in environmental management [35, 36] and it is considered an essential element in promoting desirable changes in behaviours in pursuit of sustainability [5]. Social learning, or learning that extends beyond the individual through social interaction to become situated within larger social units [36], is a widely adopted perspective within this scholarship. Social learning is manifested by collective action: forging learning partnerships, creating learning platforms, and instilling learning ethics [37]. There is very limited empirical evidence linking the promotion or enhancement of social learning to stakeholder participation [23, 36], and assumptions about the effectiveness of social learning in collaborative processes are largely untested [38].

### Outcomes

Understanding the impacts of governance and management is essential for learning and adapting decisions and actions [8]. These impacts can be identified as the results and effects of governance and management [28], collectively considered ‘outcomes’. Results are the tangible and intangible outputs of governance and management within and beyond the boundaries of the system [28, 39]. Effects are the ecological and social consequences of management and governance, including ecosystem services and enhanced livelihoods [28, 39]. Evaluating outcomes is complicated and contested. Perceptions of stakeholders have been recognized and used as an indirect measure of environmental outcomes and their potential as a source of bias is an identified concern ([40–42], see [43] for a summary). The potential presence of other variables of influence adds difficulty to correlating environmental outcomes and governance processes [44] and the potential for confounding variables and attribution issues are similarly present for social outcomes. Bennett [7] has recently challenged notions of what counts as evidence, clarifies the contributions of perceptions research, and calls for increased incorporation of evidence from social and natural sciences to enhance decisions.

We validate the relationships among participation, collaboration and learning in relation to evaluating outcomes as well as address critical gaps in knowledge about their interrelationships in environmental management and governance. using a path analysis modeling approach. Specifically, we concentrate on how participation and features of learning and collaboration influence evaluations of outcomes by the stakeholders involved. Our approach assumes that individuals’ long-term engagement in an environmental management and governance process is dependent upon their beliefs in the ability of that process to deliver desirable outcomes and that individuals engaged in the range of activities related to environmental management and governance are best-positioned to evaluate outcomes from it. We conducted the research at the individual level, collecting responses from four biosphere reserves, two in Sweden and two in Canada, for the analysis. Biosphere reserves (BRs) represent an appropriate context in which to conduct this research as members engage in governance and management activities in support of the dual aims of biodiversity conservation and sustainable development [45]; thus, they explicitly aim to achieve ecological and social outcomes.

**Fig 1:**
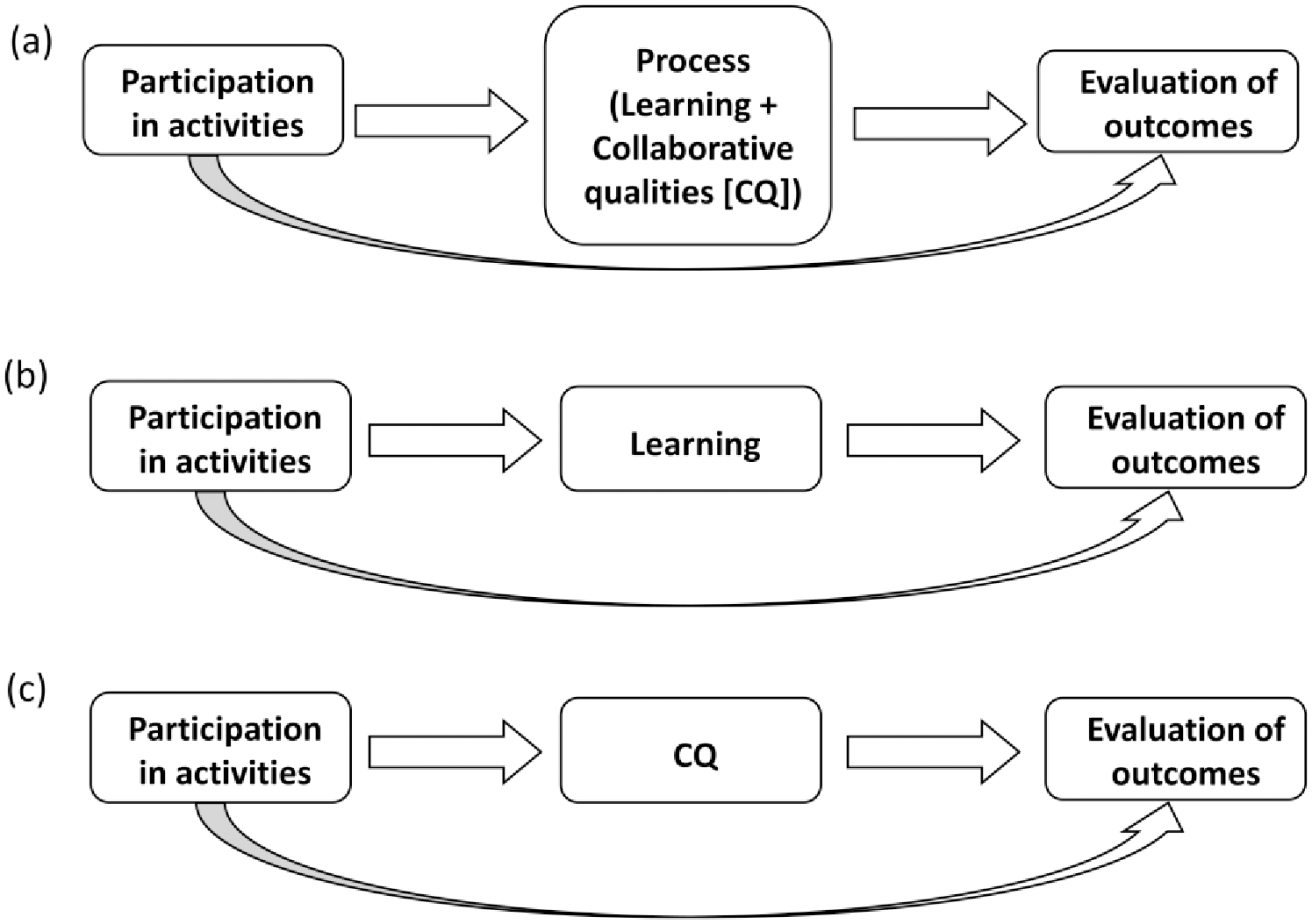
Three models used in the analysis. The first model (a) test the effect of the process generally (model 1). The process variable is then unpacked and model 2 corresponds to (b), testing the effect of learning and model 3 corresponds to (c), testing the effect of collaborative qualities. Straight arrows represent indirect effects between activities and outcomes, while curved arrows represent direct effects between them.

We investigated three potential causal pathways among participation, process (learning and collaborative qualities) and evaluating outcomes (Fig 1). The first model combines learning and collaboration into a single ‘process’ variable. In the second and third models, learning and collaborative qualities, respectively, were examined for their individual roles in the causal pathway.

## Methods

This section details the methods used for this study, including the cases, respondents, measures and analysis. The instruments used to collect data, additional detail on data preparation and the model data are available in S1–S3 Appendices. Brock University Research Ethics Board approved this research (file number 13–026).

### Cases

Four BRs were selected for study: Frontenac Arch and Georgian Bay in Canada and Kristianstads Vattenrike and Ӧstra Vätterbranterna in Sweden. These BRs were chosen based on their relative similarity in terms of size, location in high-income countries, and active governance processes. The individual was selected as the unit of analysis, and the decision to focus on these four BRs helped to minimize external variability as much as possible while providing a large enough sample of individuals engaged in management and governance interventions from the cases to conduct the analysis.

### Respondents

BR managers identified individuals in their respective BRs who were engaged in the management and governance process. These individuals were invited to participate in the study by attending a workshop and completing a questionnaire prior to participating in the workshop. Written, informed consent was provided in all cases. The final sample included 66 individuals who provided responses to all sections of the questionnaire. Respondents’ affiliations ranged from local and regional/provincial government to environmental non-governmental organizations to private business and landowners. Respondents engaged in range of activities related to management and governance of the BRs and thus their perceptions reflect their unique experiences in relation to the processes and multiple outcomes measured in this study.

### Measures

A questionnaire was administered that included questions about the specific activities in which individuals participated, quality of collaboration, learning, and evaluation of outcomes (results and effects) of the governance and management process. The items corresponding to each of these sections are provided in S1 Appendix. For activities, respondents were asked to identify specific activities in which they have participated based on seven categories: preparation of biosphere related materials for UNESCO, practical actions in the landscape, projects that involve monitoring, social events, mapping of the biosphere reserve, activities related to management and planning, and activities related to governance or decision making. The total number of identified activities was calculated for each individual.

In all other sections of the questionnaire respondents were asked to rate their agreement with provided statements on a five-point Likert response scale (*1* = *strongly disagree* and *5* = *strongly agree*). ‘Collaborative qualities’ items reflected those of established importance in the literature, such as transparency and deliberation [28, 39, 46, 47]. ‘Learning’ was measured using a typology of cognitive, normative and relational learning [39, 48]. ‘Results’ comprise evaluation of tangible and intangible products that immediately (first order) or indirectly (second order) come about from the management and governance process (see [3] for the original typology, [28, 39]). ‘Effects’ are evaluations of their consequences, consisting of ecological sustainability and enhanced livelihoods [28, 39]. Total scores were obtained for each of the five sections of the questionnaire by summing the responses to items, resulting in summary scores for each variable for each respondent. For descriptive statistics of the means and distributions of responses to each section please see S4 Appendix.

### Path analysis

Path analysis is a structural modeling approach that estimates direct and indirect effects (i.e., effects that are mediated by an intervening variable) among a set of variables [49]. Three models were examined using MPlus version 7 for Windows [50] (Fig 1). Evaluations of outcomes were specified to be the dependent variable in each model. The total number of activities each individual engaged in was used as the predictor at the start of the path analysis, and a dummy variable representing the BRs was used as a covariate to eliminate any group effects, that is, to eliminate any possible impacts membership in a particular BR might have on the analysis. Details regarding the analysis including the correlation matrix and model results are provided in S2 and S3 Appendices. The process variable was used as potential mediating variable in the first model. Thereafter, the process variable was unpacked into its component parts: learning and collaborative qualities. Learning was used as a potential mediating variable in the second model, and collaborative qualities in the third model (Fig 1b and 1c, respectively). Thus, models 2 and 3 were used to explore the relative contributions of learning and collaborative qualities to the relationship between activities and outcomes. The goal of the analysis was to understand the indirect effects (i.e., effects of activities on outcomes through process/learning/collaborative qualities) and direct effects (i.e., effects of activities on outcomes). The significance of indirect effects was examined through 95% confidence intervals (CIs) using maximum likelihood estimators with 1000 bootstrap samples, such that any effects that did not include 0 in the CI were considered to be significant.

## Results

**Fig 2:**
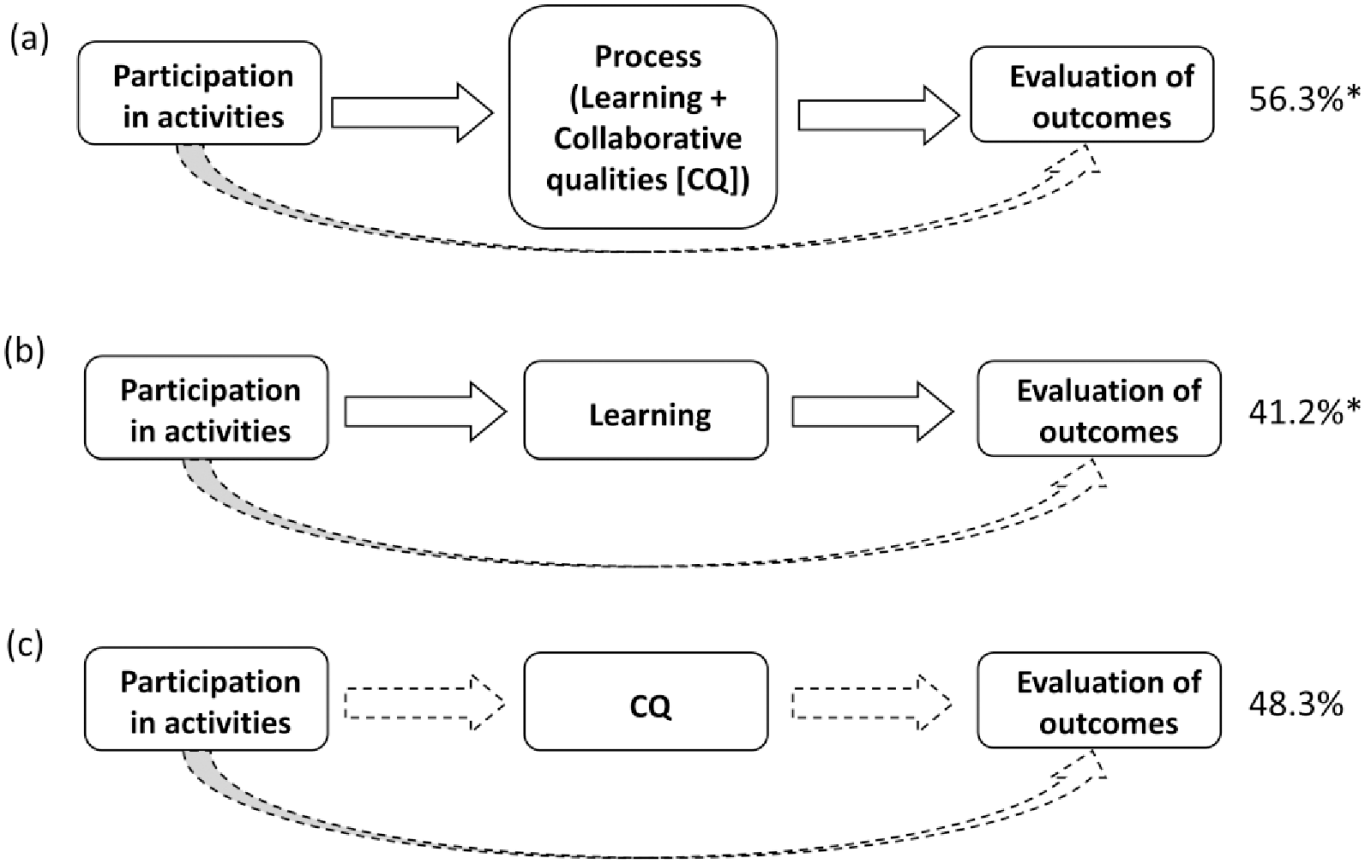
Variance explained in indirect effects in the three models (percentage values on right side of figure). Solid arrows indicate significant effects; dashed arrows represent non-significant relationships. *Significant indirect effect.

The first model examined the relationship between activities, process (i.e., composite of learning and collaborative qualities) and evaluations of outcomes (Fig 2a). Activities and process together explained 56.3% (*p* < .001) of variance in evaluations of outcomes and activities alone explained 22.6% (*p* = .002) of variability in process. The direct effects of activities on evaluations of outcomes was not significant but the indirect effect of activities to outcomes through process was, such a higher level of participation was related to higher scores on Process measures and in turn, greater scores for outcomes.

In the alternative models examined, the process variable was substituted with either learning (model 2) or collaborative qualities (model 3) (Fig 2b and 2c). Activities explained 19.5% (*p* = .007) of variance in learning and 41.2% (*p* < .001) of variance in evaluations of outcomes. Similar to the results of the first model, only the indirect effect of activities on evaluations of outcomes (i.e., through learning) was significant. In the third model, activities explained 23.4% of variance in collaborative qualities (*p* = .019) and 48.3% of variance in evaluations of outcomes (*p* < .001). However, neither the direct nor indirect effects were significant. Thus, it appears that effects of activities on evaluations of outcomes is influenced more by the path through learning compared to quality of collaborations. It is worth noting that no group effects related to membership in a particular BR were found in any model.

## Discussion

### Evidence to support the importance of a quality management and governance process

Insights derived from the analysis unveil the dynamics ‘on the ground’ as perceived by stakeholders engaged in management and governance in BRs. As captured in the first model (Fig 2a), participation in more activities led to higher rating of process variables and, in turn, more positive evaluation of social and ecological outcomes. Our finding gives insight into previous studies where participation in more management and governance activities is assumed to lead to improved decisions and strongly depends on the quality of the process [23]. This finding regarding the importance on the quality of the process itself is not surprising. However, the question of what constitutes a quality process has given rise to syntheses of principles and attributes in resource and environmental management (e.g., [23, 51–53]), as well as in environmental governance and resilience scholarship (e.g., [12, 54]). Our findings complement these insights from qualitative studies and confirm, empirically, the combination of collaboration and learning as foundational to better perceptions among stakeholders about outcomes from environmental management and governance. We were able to account for a substantial amount of the variance of evaluated outcomes (56.3%) in our first model using the combined ‘process’ variable.

The findings resonate with governance and management efforts more broadly. The BRs can be considered as “early movers and motivators” in addressing the stewardship challenge [2: 7373] and thus provide an excellent context in which to conduct this research. Commenting on management and governance effectiveness oversteps our results, but our findings contribute two valuable insights. First, we broaden the basis for evidence about outcomes beyond a single source, such as a BR manager, to include stakeholders. Second, we are able to enhance knowledge regarding the extent of variance of outcomes explained by participation and process variables. Insights into these approaches almost exclusively come from qualitative case studies and reviews of the literature (e.g., [11, 16]), but see Stoll-Kleeman et al. [55] and Schultz et al. [21] for exceptions that revealed a belief by managers about the importance of community participation, especially in association with acceptance of the organization/designation and conservation program success. Although tremendous value is derived from the richness of studying cases, and context is clearly important, several limitations have been observed in the literature to date (e.g., inconsistent conceptualization, imprecise definitions, lack of measurements), that hamper the comparability of research and the ability to draw causal inferences [16, 39]. Our study has directly addressed these limitations. The modeling and statistical analysis employed here offer tools for further application in adaptive governance as well as a broader suite of approaches to environmental collective action.

### Learning contributes more than collaboration to perceived outcomes

Deconstructing the first model and examining the effects of learning and collaborative qualities separately (models 2 and 3, Fig 1) showed that participation in more activities contributes to greater learning scores but not to better perceptions of collaborative qualities. Therefore, the effects of activities on evaluating outcomes appear to be driven by learning rather than quality of collaboration. However, since the overall model (Fig 2a) where the process variable included both learning and collaborative qualities is significant, these analyses highlight the relative importance of each process sub-variable (i.e., learning seems more important when explaining effects of activities). These findings enrich the dialogue among scholars about how a group of actors come to address collective action problems when managing linked systems of people and nature. First, the frequency of engagement in activities is important: those who engaged in more activities tended to learn more and provide more positive evaluations of outcomes. Second, and related specifically to the group processes, collaborating in a ‘quality’ process is seen as the means to addressing collective action problems (e.g., [31, 41, 56]). But also, addressing collective action problems is reliant upon learning, and learning comes about through a ‘quality process’ (e.g. [5, 37, 57, 58]). The pathways revealed through our analysis augment these existing causal presumptions and align with recent assertions by Lubell [59, 60] that solving collective-action problems requires complex institutional systems that facilitate key processes of learning, cooperation and distribution of benefits and costs.

### Shedding light on conceptual and analytical considerations

Conceptual tensions and analytical complications surround research about approaches to environmental management and governance in relation to outcomes. We elected to use self-reported information by the individuals who participated in the activities of the BRs. Our choice is predicated upon the logic that the individuals involved in the initiative are best positioned to reflect upon the process as well as evaluate the outcomes of interest (see S1 Appendix), and this choice is supported by research that shows that stakeholders with different experiences and knowledge bring different perceptions of the same resources [61]. We statistically control for BR level effects with regards to processes and outcomes so that analysis throughout is at the individual level. Moreover, we utilize a multi-item approach for rating outcomes as opposed to a single-item effectiveness measure, to hedge against inflated perceptions by stakeholders as found by Selin et al. [6]. These decisions in our analytical approach were made to enable a focus on the features influencing evaluation of outcomes by the stakeholders involved.

Utilizing independent measures of environmental outcomes is complementary to our approach and a laudable ideal (see [43] for a recent example), as is pursuing innovative research designs that present opportunities to limit confounding variables (e.g., [44]). Enthusiasm for independent measures must be tempered with awareness of the inherent difficulties in understanding and attributing environmental [9] as well as social impacts. The indirect path in our first model is consistent with findings in collaborative environmental management of positive evaluations of an initiative by active participants [6]. Although a multi-item approach was utilized to enhance the validity of the measures and potentially counter inflation of evaluations, it is not possible to rule out the ‘halo effect’, which occurs when there are high levels of trust among individuals, inflating perceptions of their collective impact [62]. The indirect path is also consistent with the broader phenomena of cognitive dissonance where respondents engaging in more intensive endeavours exaggerate the associated positive outcomes [63]. The direct path from participation to evaluation of outcomes was not statistically significant. This opens interesting opportunities to expand the model and probe additional features which may be exerting influence on the evaluation of outcomes, such as personal attributes or specific institutional and political conditions within which governance occurs. Finally, we utilized one portion of a more extensive dataset in which individuals are nested within BRs. Attaining and utilizing a larger and longitudinal dataset opens a host of possibilities for conceptual and analytical advancement. Specifically, aggregated variables such as outcomes could be tested as separate variables (i.e., results and effects); an understanding of feedbacks could be developed; and, analysis could be conducted at a level (individual, BR) and/or at multiple levels, capturing multi-level interactions.

### Implications for practice and policy

Our findings suggest that stakeholder engagement in multiple activities influences their experiences in environmental management and governance and, in turn, their evaluation of the outcomes. It highlights the importance of a positive experience in environmental stewardship efforts for continued engagement [64] and specifically identifies the critical role of learning [65]. Participation by volunteers in particular has been considered essential to the functioning of environmental stewardship programs [64,66], with learning highlighted as an important motivator for engagement. Accordingly, practices that create opportunities for engagement of stakeholders in multiple activities and for learning to occur are likely to improve outcomes as evaluated by those stakeholders. As Bennett [7: 584] reminds us, “it is positive perceptions, not just objective scientific evidence of effectiveness, that ultimately ensure the support of local constituents thus enabling the long-term success of conservation”.

Our research offers first steps to in a larger effort to identify important practical guidelines that enhance engagement in, and outcomes from, environmental stewardship. However, it is important to note that the relatively small sample size, while certainly adequate for the models tested here would benefit from a large sample to improve both the power of the analysis and the ability to test relationships among more variables. Accordingly, further research to build a nuanced understanding of participation in different kinds of activities, types of learning and specific collaborative qualities that are most influential on perceived outcomes, and how virtuous cycles of engagement are created, is needed. Research of this kind, when undertaken with a very large sample of environmental stewardship stakeholders in a range of contexts, can provide guidance for practitioners in terms of how to encourage participation and nurture a positive management and governance process to enhance social and ecological outcomes.

## Conclusions

Participation, collaboration and learning are advanced as important features of approaches to contemporary environmental management and governance. The findings from our research afford evidence that their importance can be empirically determinable in relation to the evaluation of outcomes. Our work to untangle the dynamics and relative contributions of these essential features to evaluation of outcomes advances the understanding and importance of stakeholder perceptions [7], empirical connections between interventions with outcomes [9], and inner-workings of social-ecological governance approaches taking up the stewardship challenge [2]. We show here that the scope of inquiry should be extended beyond collaboration [60, 67] to a broader suite of approaches for managing and governing social-ecological systems. Insights about pathways to address collective action problems appear somewhat fragmented in the literature, and further research is required to close this gap and/or discern multiple processes facilitating management and governance arrangements [59, 60]. Efforts to advance our understanding of these pathways will greatly benefit from an expanded acceptance of what constitutes evidence (i.e., perception as we use it in this analysis), as well as more effective use of qualitative and quantitative measures from natural and social sciences [7]. Ultimately, such a research agenda may enable us to empirically connect management and governance interventions to outcomes, and therefore, offer 1) an evidence-based argument for necessary changes [8, 9]; and 2) a richer understanding of the features influencing the stakeholder views that are critical for long-term success [7].

## Supporting information

S1 Appendix Data collection instrument. (DOCX)

S2 Appendix Additional detail on data preparation. (DOCX)

S3 Appendix Additional detail on data analysis and preparation. (DOCX)

S4 Appendix Descriptive statistics. (DOCX)
