# Supplementary material for: How do environmental governance processes shape evaluation of outcomes by stakeholders? A causal pathways approach
Source: PLoS One. 2017 Sep 25;12(9):e0185375. doi: 10.1371/journal.pone.0185375 (PMC5612751; doi:10.1371/journal.pone.0185375)
Supplement: S1 Appendix — (DOCX) [file pone.0185375.s001.docx]

*Activities*

1. Below, we have listed categories of activities common in biosphere reserves. Please consider each category and identify specific activities in which you have participated.

Note: Please focus on activities connected to the biosphere reserve organization / management and list as many as you can think of in the space provided below each category.

1. Preparation of biosphere reserve related materials for UNESCO (e.g. application to become a biosphere reserve, periodic review):
2. Practical actions in the landscape (e.g., restoration project, operation of a farm):
3. Projects that involve monitoring (e.g., biological inventories):
4. Social events (e.g., festivals):
5. Mapping of the biosphere reserve:
6. Activities related to management and planning (e.g., management plan):
7. Activities related to the governance or decision making (e.g., steering committee, council, advisory board):

*Learning*

*In this section of the questionnaire we are interested in understanding how the {name of BR} has influenced you****.*** *Please note that ‘BR’ refers to Biosphere Reserve.*

1. For each statement below, please indicate the extent to which you agree or disagree:

(Circle the appropriate number on the scale from strongly disagree (1) to strongly agree (5))

|  | Strongly  Disagree |  |  |  | Strongly Agree |
| --- | --- | --- | --- | --- | --- |
| My understanding of the ecological conditions of the BR has increased due to my involvement in the BR management process. | 1 | 2 | 3 | 4 | 5 |
| My understanding of the social conditions of the BR have increased due to my involvement in the BR management process. | 1 | 2 | 3 | 4 | 5 |
| My knowledge of the problems and challenges of the BR has increased due to my involvement in the BR management process. | 1 | 2 | 3 | 4 | 5 |
| My knowledge of the opportunities of the BR has increased due to my involvement in the BR management process. | 1 | 2 | 3 | 4 | 5 |
| A majority of my current knowledge about the landscapes of the BR comes from my involvement with BR management. | 1 | 2 | 3 | 4 | 5 |
| The BR management process has helped me understand the perspective of others. | 1 | 2 | 3 | 4 | 5 |
| The BR management process has become more important over time. | 1 | 2 | 3 | 4 | 5 |
| My views on the BR have led me to act in surprising or new ways. | 1 | 2 | 3 | 4 | 5 |
| My views on the BR are similar to those of others involved in the BR management process. | 1 | 2 | 3 | 4 | 5 |
| Over time, the process has changed my view on which goals should steer the management of the area. | 1 | 2 | 3 | 4 | 5 |
| Involvement in the BR has enhanced my cooperation/coordination with other individuals and groups/organizations *within* the BR. | 1 | 2 | 3 | 4 | 5 |
| Involvement in the BR has enhanced my cooperation/coordination with other individuals and groups/organizations *outside* the BR. | 1 | 2 | 3 | 4 | 5 |
| Involvement in the BR has enhanced my communication with other individuals and groups/organizations *within* the BR. | 1 | 2 | 3 | 4 | 5 |
| Involvement in the BR has enhanced my communication with other individuals and groups/organizations *outside* the BR. | 1 | 2 | 3 | 4 | 5 |

*Collaborative Qualities*

*In this section we are interested in the process of collaborative management and governance in {BR name}. That is, the activities and practices undertaken as a group to manage and govern the biosphere reserve.*

1. For each statement below, please indicate the extent to which you agree or disagree regarding the collaborative process (including both management and governance):

(Circle the appropriate number on the scale from strongly disagree (1) to strongly agree (5))

|  | Strongly  Disagree |  |  |  | Strongly  Agree |
| --- | --- | --- | --- | --- | --- |
| The process has been characterized by widely differing opinions regarding the view on problems, goals and strategies. | 1 | 2 | 3 | 4 | 5 |
| The process has been characterized by deep conflict (i.e., conflict that is strong and enduring) between the actors involved. | 1 | 2 | 3 | 4 | 5 |
| The process gives all actors equal opportunities to state their opinion and to influence the outcome. | 1 | 2 | 3 | 4 | 5 |
| The process is transparent. | 1 | 2 | 3 | 4 | 5 |
| The process encourages reasonable and constructive discussions. | 1 | 2 | 3 | 4 | 5 |
| The process encourages new solutions. | 1 | 2 | 3 | 4 | 5 |
| The process is characterized by trust and confidence among the involved actors. | 1 | 2 | 3 | 4 | 5 |
| Over time, the process has changed my view on how management of the BR should be best organized. | 1 | 2 | 3 | 4 | 5 |
| Some individuals have been/are particularly important for facilitating learning from our activities and discussions. | 1 | 2 | 3 | 4 | 5 |
| Some actors have had/continue to have a disproportionately large influence on decisions (e.g. have tended to dominate decision making processes). | 1 | 2 | 3 | 4 | 5 |

*Results*

*In this section we would like you to assess the results and the effects of the process of collaborative management and governance in {BR name} up until now. This includes the activities and practices undertaken as a group to manage and govern the {name of BR}.*

1. For each statement below, please indicate the extent to which you feel the process of collaborative management and governance has resulted in:

(Circle the appropriate number on the scale from strongly disagree (1) to strongly agree (5))

|  | Strongly  Disagree |  |  |  | Strongly Agree |
| --- | --- | --- | --- | --- | --- |
| Enhanced legitimacy (i.e. a more transparent, equitable and fair processes for decision-making) | 1 | 2 | 3 | 4 | 5 |
| Greater support by those not involved in the process of collaborative management and governance for BR decisions | 1 | 2 | 3 | 4 | 5 |
| New co-operative undertakings with partners within the geographical boundary of the BR | 1 | 2 | 3 | 4 | 5 |
| New co-operative undertakings with partners beyond the geographical boundary of the BR | 1 | 2 | 3 | 4 | 5 |
| Ability to engage with a broader set of issues and challenges within the BR (i.e., increasing scope and mandate) | 1 | 2 | 3 | 4 | 5 |
| Greater efficiency of the group involved in BR governance and management in making decisions and responding to issues | 1 | 2 | 3 | 4 | 5 |
| Greater flexibility of the group involved in BR governance and management with how challenges are addressed | 1 | 2 | 3 | 4 | 5 |
| Informal agreements of how to address an issue | 1 | 2 | 3 | 4 | 5 |
| Novel approaches to solving problems within the group involved in BR governance and management | 1 | 2 | 3 | 4 | 5 |
| Fostering dialogue between different interests | 1 | 2 | 3 | 4 | 5 |

*Effects*

1. Please indicate the extent to which you feel the process of collaborative management and governance has effected:

(Circle the appropriate number on the scale from strongly disagree (1) to strongly agree (5) for each row)

|  | Strongly  Disagree |  |  |  | Strongly Agree |
| --- | --- | --- | --- | --- | --- |
| Ecological sustainability within the BR (e.g., biodiversity protection, maintaining ecosystem services) | 1 | 2 | 3 | 4 | 5 |
| Ecological sustainability beyond the BR (e.g., biodiversity protection, maintaining ecosystem services) | 1 | 2 | 3 | 4 | 5 |
| Enhancements in wellbeing for those living within the geographical boundary of the BR | 1 | 2 | 3 | 4 | 5 |
| Decreases in vulnerability for those living within the geographical boundary of the BR | 1 | 2 | 3 | 4 | 5 |
| More sustainable resource use within the BR | 1 | 2 | 3 | 4 | 5 |
| Improvements in human capital (e.g., skills, capacity) by for those living within the geographical boundary | 1 | 2 | 3 | 4 | 5 |
| Improvements in social capital (e.g., trust, relationships, working together) by those involved in the collaborative management and governance of the BR. | 1 | 2 | 3 | 4 | 5 |
| Improvements in physical capital (e.g., infrastructure, communication systems, community facilities) by those involved in the collaborative management and governance of the BR. | 1 | 2 | 3 | 4 | 5 |
| Stimulating sustainable economic development (e.g. improvements in economic opportunities for individuals and communities such as employment opportunities, income improvements) within the geographical boundary of the BR. | 1 | 2 | 3 | 4 | 5 |
