# Supplementary material for: How do environmental governance processes shape evaluation of outcomes by stakeholders? A causal pathways approach
Source: PLoS One. 2017 Sep 25;12(9):e0185375. doi: 10.1371/journal.pone.0185375 (PMC5612751; doi:10.1371/journal.pone.0185375)
Supplement: S2 Appendix — (DOCX) [file pone.0185375.s002.docx]

**Additional detail on data preparation**

In the final sample of 66 respondents, 51 responded to all items of the questionnaire. In the other 15 cases, respondents missed one or several items on the questionnaire. In order to maximize statistical power, total scores for those responding to 80% or more of each section were estimated by multiplying the mean of the items by the total number of items within each section where data were missing to be able to include more respondents in the final dataset. An additional measure taken to further maximize statistical power: reducing the number of variables in the path analysis by creating composite variables of ‘process’ and ‘outcomes’ by taking the average of the standardized total scores for relevant variables (i.e., learning and collaborative qualities for process and results and effects for outcomes). This approach gives equal weighting to both variables that comprise the composites and does not favour any variable with a higher number of items. Thus, for the final analysis, the data were summarized in three variables (Fig. 1a): activities (total count of activities reported), process (composite of learning and collaborative qualities) and outcomes (composite of results and effects).

These variables were screened for outliers (±3SD) and checked for normality. No data were removed as a result of the screening. Bivariate correlations between variables of interest were then examined. As total number of activities were not normally distributed, Spearman’s rho coefficients were used to examine relationships with this variable; whereas, relationships between process and outcome variables was examined using Pearson coefficients. Data preparation was carried out using SPSS 21.0 statistical package [1].

**References**

1. IBM Corp. *IBM SPSS Statistics v. 21.0 for Windows*. Armonk, NY: IBM Corp; 2012.
