# Supplementary material for: How do environmental governance processes shape evaluation of outcomes by stakeholders? A causal pathways approach
Source: PLoS One. 2017 Sep 25;12(9):e0185375. doi: 10.1371/journal.pone.0185375 (PMC5612751; doi:10.1371/journal.pone.0185375)
Supplement: S3 Appendix — (DOCX) [file pone.0185375.s003.docx]

**Additional detail on data analysis and results**

In order to examine relationships between variables prior to creating the composite variables (process and outcomes), bivariate correlations were computed using the sample of participants who responded to at least 80% of items of the questionnaire (S3A Table). Activities were positively correlated with all measures, such that individuals who participated in more activities also had higher scores on other measures, with the exception of effects. When bivariate correlations were computed using the composite variables, the total number of activities did not significantly correlate with the composite outcome measure, however, the a priori hypothesis that process mediates the relationship between activities and outcomes, and the presence of a significant relationships with results (i.e., part of the composite measure) and process composite (as well as learning and collaborative qualities separately) were considered sufficient reasons to proceed with the path analysis.

**S3A Table.** Zero-order correlations between measures including individuals responding to at least 80% of items in the questionnaire (*N* = 66).

|  | *Process*  *(composite)* | *Outcome*  *(composite)* | CQ | Learning | Results | Effects |
| --- | --- | --- | --- | --- | --- | --- |
| Activities | .54** | .23 | .39** | .41** | .35** | .02 |
| Collaborative Qualities (CQ) | .75** | .57** |  | .19 | .63** | .40** |
| Learning | .80** | .42** |  |  | .46** | .28* |
| Results | .70** | .90** |  |  |  | .61** |
| Effects | .43** | .89** |  |  |  |  |
| *Process (composite)* |  | .64** |  |  |  |  |
| * *p*<.05 ** *p*< .01 |  |  |  |  |  |  |

**Model results of the path analysis**

Results of the path analysis for the three models are summarized in S3B Table. As all of the models were just-identified, there were no model fit indices to compare across the models. Activities were not normally distributed, so robust maximum likelihood parameter estimates [MLR estimator option in MPlus 9, 1] were calculated. Thus, standardized coefficients for the predictors (i.e., beta values) and unstandardized effects are reported. It should be noted that although the total effect was not significant, the decomposed effects are still meaningful [see 2 for review].

**S3B Table.** Summary of coefficients and effects for the three path analysis models conducted.

|  | **Model 1** | | | | **Model 2** | | | **Model 3** | | |
| --- | --- | --- | --- | --- | --- | --- | --- | --- | --- | --- |
|  | Process | Outcomes | | | Learning | Outcomes | | CQ | Outcomes | |
| Predictor | Activities | Activities | Process | Activities | | Activities | Learning | Activities | Activities | CQ |
| B-weights | 0.08 | -0.04 | 0.74 | 0.85 | | -0.03 | 0.06 | 0.25 | <0.01 | 0.09 |
| SE | 0.02 | 0.02 | 0.11 | 0.19 | | 0.02 | 0.02 | 0.15 | 0.02 | 0.02 |
| *Β* | 0.43 | -0.18 | 0.62 | 0.48 | | -0.12 | 0.44 | 0.20 | <0.01 | 0.49 |
| *p* values | .001 | .068 | <.001 | <.001 | | .264 | <.001 | .083 | .983 | <.001 |
| Total effects | – | 0.019 | | – | | 0.020 | | – | 0.023 | |
| Direct effects | – | -0.042 | | – | | -0.027 | | – | <0.01 | |
| Indirect effects | – | 0.060** | | – | | 0.047** | | – | 0.023 | |
| 95% CI | – | 0.026 - 0.117 | | – | | 0.018 - 0.090 | | – | -0.006 – 0.066 | |

***p<.01*

**References**

1. Muthén L K and Muthén B O 2013 *MPlus User’s Guide 7^th^ Ed* .Los Angeles: Muthén & Muthén; 2013.
2. Loeys T, Moerkerke B and Vansteelandt S. A cautionary note on the power of the tests for indirect effect in mediation analysis. *Frontiers in Psychology* 2015 Jan; 5: 1–8.
