# Supplementary material for: How do environmental governance processes shape evaluation of outcomes by stakeholders? A causal pathways approach
Source: PLoS One. 2017 Sep 25;12(9):e0185375. doi: 10.1371/journal.pone.0185375 (PMC5612751; doi:10.1371/journal.pone.0185375)
Supplement: S4 Appendix — (DOCX) [file pone.0185375.s004.docx]

**Summary statistics for model variables**

**S4 Table**. Summary statistics for model variables

| Variable | Mean | Standard deviation | Min | Max |
| --- | --- | --- | --- | --- |
| Activities^1^ | 5.79 | 4.08 | 0 | 17 |
| Collaborative qualities^2^ | 33.15 | 4.49 | 22 | 41 |
| Learning^3^ | 46.05 | 6.85 | 30 | 59 |
| Results^4^ | 44.08 | 7.31 | 31 | 60 |
| Effects^5^ | 29.68 | 6.57 | 18 | 43 |

^1^ Number of activities each participant indicated they engaged in

^2^ Sum of responses to 10 items, using a 5-point Likert scale (1=strongly disagree; 5= strongly agree)

^3^ Sum of responses to 14 items, using a 5-point Likert scale (1=strongly disagree; 5= strongly agree)

^4^ Sum of responses to 10 items, using a 5-point Likert scale (1=strongly disagree; 5= strongly agree)

^5^ Sum of responses to 9 items, using a 5-point Likert scale (1=strongly disagree; 5= strongly agree)
